# Transient Coronary Microvascular Dysfunction in Septic Shock

**DOI:** 10.1016/j.jaccas.2025.103611

**Published:** 2025-06-11

**Authors:** Samantha Lörstad, Sara Tehrani, Christina Ekenbäck, Kambiz Shahgaldi, Per Åstrand, Johannes Aspberg, Jonas Persson

**Affiliations:** a Division of Internal Medicine, Department of Clinical Sciences, Danderyd University Hospital, Karolinska Institutet, Stockholm, Sweden; b Division of Cardiovascular Medicine, Department of Clinical Sciences, Danderyd University Hospital, Karolinska Institutet, Stockholm, Sweden

**Keywords:** acute coronary syndrome, atherosclerosis, cardiac risk, cardiomyopathy, circulation, coronary angiography, coronary circulation, fractional flow reserve, myocardial ischemia, perfusion

## Abstract

**Background:**

Sepsis is a dysregulated hyperinflammatory response to infection that leads to widespread endothelial damage with impaired microcirculation and organ perfusion. The extent of coronary microvascular dysfunction in sepsis remains largely unexplored.

**Case Summary:**

We present a case of pneumococcal pneumonia complicated by septic shock, where coronary microvascular function was invasively assessed using the thermodilution technique. We observed transient dysfunction in the coronary microcirculation during the transition from the acute to recovery phases of septic shock.

**Discussion:**

This transient dysfunction does not necessarily signify full coronary microvascular recovery because acute inflammation may lead to long-term changes in vascular structure. This case provides novel insights into cardiac microvascular involvement in sepsis and highlights the importance of further research on its impact on patient outcomes.

**Take-Home Messages:**

Sepsis can cause coronary microvascular dysfunction, even with preserved left ventricular ejection fraction. Sepsis-induced coronary microvascular dysfunction can resolve during the recovery phase.

## Visual Summary

**Figure:**
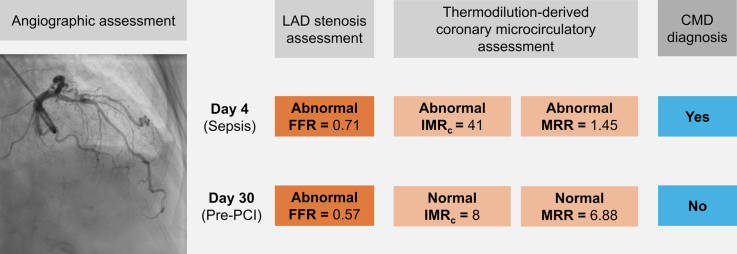

Sepsis triggers widespread hyperinflammatory changes in the microcirculation, leading to endothelial dysfunction, intravascular thrombosis, compromised barrier function, dysregulated vasomotor tone, obstructed blood flow, and tissue edema.^1^ Although coronary microvascular changes have been studied in animal sepsis models, in vivo investigations in humans present technical challenges, leaving the prevalence and impact of sepsis-related coronary microvascular dysfunction (CMD) poorly understood.

**Take-Home Messages**

• This case highlights that sepsis can lead to CMD, even in the presence of preserved LVEF.
• Sepsis-induced CMD can resolve during the recovery phase.

## History of Presentation

In December 2023, a 67-year-old Caucasian female presented to Danderyd University Hospital in Stockholm, Sweden reporting a 2-day history of fever, chills, oliguria, and right-sided chest pain radiating to the scapular region. She was afebrile, alert, and oriented, with an oxygen saturation of 88% on room air and a respiratory rate of 30 breaths/min. Her blood pressure was 94/41 mm Hg, and there was no lower extremity edema. An electrocardiogram revealed sinus tachycardia (110 beats/min) with normal QRS complexes and no ST-T wave changes.

Within 4 hours of admission, oxygen therapy at 4 L/min improved her oxygen saturation to 94%. She required 3 L of isotonic fluid to stabilize recurrent hypotension. Initial laboratory investigations revealed elevated inflammatory markers, elevated lactate, mild anemia, and acute kidney injury (Table 1). A computed tomography scan of the chest revealed right-sided pulmonary infiltrates. The patient was started on intravenous cefotaxime and an increased dose of oral corticosteroids and was then admitted to the intermediate medical care unit.

**Table 1:** Daily Clinical Parameters and Laboratory Values

	Day 1	Day 2	Day 3	Day 4	Reference Range
Severity scores					
SOFA score	7	10	5	3	0-24
APACHE II	—	24	—	—	0-71
Laboratory values					
Hemoglobin, g/dL	11.4	10.3	9.8	8.3	11.7-15.3
White blood cell count, ×10^3^/μL	4.1	1.7	6.2	11.0	3.5-8.8
Platelet count, ×10^3^/μL	217	178	206	177	165-387
Hematocrit, %	34	30	29	24	36-48
Creatinine, mg/dL^a^	6.55	4.40	2.36	1.46	<1.02
Estimated GFR, mL/min/1.73 m^2^	7	11	20	34	>60
Bilirubin, mg/dL	0.94	0.64	0.58	0.58	<1.5
Procalcitonin, ng/mL	—	24	—	—	<0.5
C-reactive protein, mg/L	490	489	490	280	<3
Hs-cTnT, ng/L^b^	—	1) 24 2) 43	(3) 19	—	<15
NT-proBNP, pg/mL^c^	—	13,900	—	—	<222
Arterial lactate, mmol/L	3.2	1.9	1.8	0.8	0.5-2.3

Apache II = Acute Physiology and Chronic Health Evaluation II; GFR = glomerular filtration rate; hs-cTnT = high-sensitivity cardiac troponin T; NT-proBNP = N-terminal pro–B-type natriuretic peptide; SOFA = Sequential Organ Failure Assessment.

a The patient’s habitual creatinine level before hospitalization was 0.96 mg/dL (estimated GFR, 58 mL/min/1.73 m^2^).

b For hs-cTnT, measurements were sampled at 3-hour intervals: 1) day 2, 19:00; 2) day 2, 22:00; 3) day 3, 01:00.

c NT-proBNP was measured on day 2 at 19:00.

On day 2, blood cultures identified *Streptococcus pneumoniae.* Still requiring oxygen therapy at 1 L/min, the patient experienced fatigue and confusion, hypotension (80/40 mm Hg), and reduced urine output unresponsive to fluids. Norepinephrine was initiated to maintain mean arterial pressure above 65 mm Hg. Elevated high-sensitivity cardiac troponin T (hs-cTnT) and N-terminal pro–B-type natriuretic peptide (NT-proBNP) levels were noted (Table 1).

On day 3, after discontinuing norepinephrine, the patient developed normofrequent (60-100 beats/min) atrial fibrillation while remaining hemodynamically stable.

The patient in this report participated in the COMTESS (COronary Microcirculation and Troponin Elevation in Severe Sepsis; NCT06294730) study. This observational study investigates the relationship between elevated hs-cTnT levels (>15 ng/L) within 48 hours of sepsis onset and invasive coronary microvascular indices measured between days 3 and 10 after achieving clinical stability.

## Past Medical History

The patient had hypertension, polymyalgia rheumatica treated with oral corticosteroids, hypothyroidism managed with levothyroxine, and currently untreated multiple myeloma. Her body mass index was 24 kg/m^2^, and she had stopped smoking 15 years previously. She denied experiencing angina pectoris symptoms.

## Differential Diagnosis

This case involves pneumococcal pneumonia complicated by septic shock. The patient exhibited significantly elevated hs-cTnT levels and a markedly elevated NT-proBNP level during vasoactive therapy, suggestive of acute myocardial injury and dysfunction. Differential diagnoses include type 1 or type 2 myocardial infarction, takotsubo syndrome, and sepsis-related myocardial injury.

## Investigations

On day 3, transthoracic echocardiography showed a normal-sized left ventricle with a mass of 75 g/m^2^, mild septal hypertrophy (12 mm), and no regional wall motion abnormalities. The left ventricular ejection fraction (LVEF) was preserved (50%-55%), although forward output was decreased (left ventricular [LV] stroke volume of 33 mL/m^2^). LV global longitudinal strain was reduced (−13%; reference: <−18%), and there was evidence of LV-arterial uncoupling (ratio of arterial to ventricular elastance [Ea/Ees] = 2.6), indicating a mismatch between the left ventricle and arterial elastance. LV filling pressure was estimated as normal on the basis of the ratio of peak mitral inflow velocity during early diastole to peak early diastolic mitral annular velocity (E/e′) and normal systolic pulmonary artery pressure. The right ventricle was of normal size with preserved systolic function, and both atria were enlarged. Doppler measurements indicated moderate secondary mitral and tricuspid valve regurgitation, with normal systolic pulmonary artery pressure.

**Figure 1:**
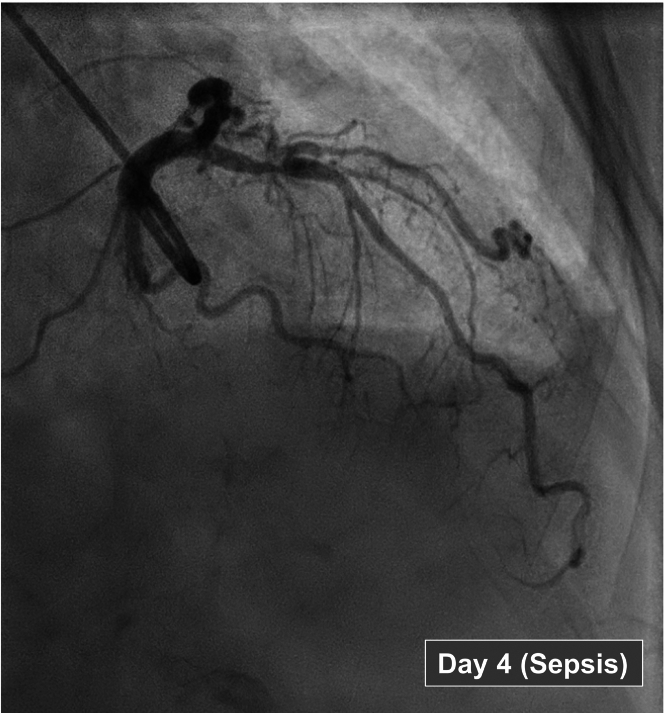
Coronary Angiography of the Left Anterior Descending Artery on Day 4 Following Sepsis Symptom Onset

**Figure 2:**
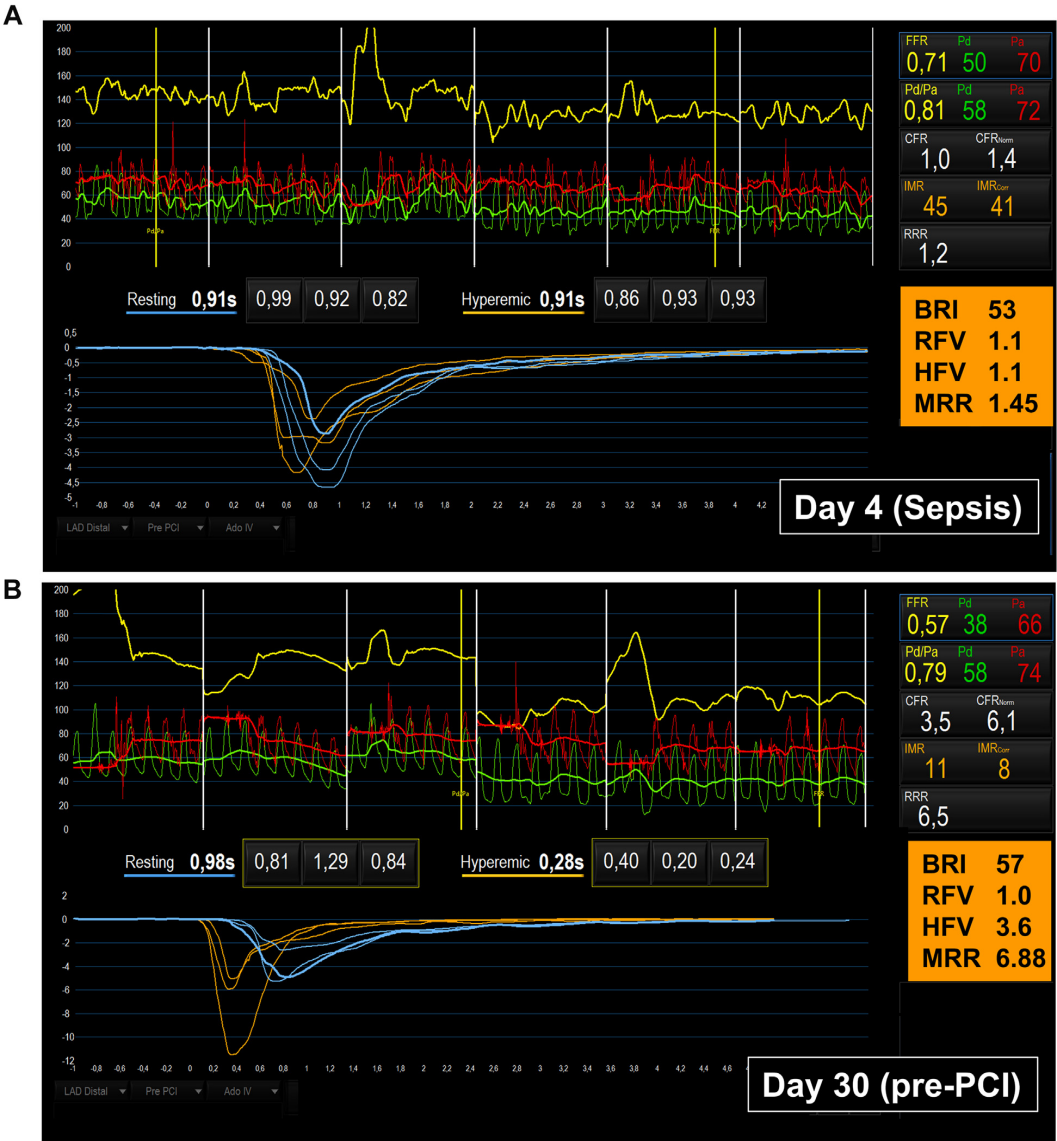
Coronary Pressure and Flow Velocity Thermodilution Measurements Measurements on (A) day 4 (sepsis phase) and (B) day 30 (recovery phase, before percutaneous coronary intervention [pre-PCI]). Measurements of resting and hyperemic flow along with computed resistance indices: fractional flow reserve (FFR), coronary flow reserve (CFR), index of microcirculatory resistance (IMR), corrected index of microcirculatory resistance (IMR_corr_), baseline resistance index (BRI), resting flow velocity (RFV), hyperemic flow velocity (HFV), and microvascular resistance reserve (MRR). Pa = aortic pressure; Pd = distal coronary pressure; RRR = resistive reserve ratio.

On day 4, coronary angiography revealed an obstructive lesion in the proximal left anterior descending (LAD) artery (Figure 1), confirmed by a fractional flow reserve (FFR) of 0.71 (normal >0.80), indicating significant epicardial obstruction (Figure 2A). To evaluate myocardial microvascular function, the thermodilution technique was used. This technique, previously used in patients with coronary artery disease, involves injecting a saline bolus into a coronary artery and measuring coronary pressure and saline transit time. This process enables the assessment of microvascular function both at rest and during adenosine-induced hyperemia.^2^ By using this method, coronary flow reserve and measurements of microvascular resistance were calculated, including the baseline resistance index, the corrected index of microcirculatory resistance (IMR_c_), and microvascular resistance reserve (MRR) (Table 2).^2^, ^3^, ^4^

**Table 2:** Summary of Coronary Flow and Microcirculatory Resistance Indices and Formulas

Flow Index	Formula	Explanation
Index of microcirculatory resistance (IMR)	$P_{dHyp}\times {Tmn}_{Hyp}$	Hyperemic microcirculatory resistance
Corrected index of microcirculatory resistance (IMR_C_)	$P_{aHyp}\times {Tmn}_{Hyp}\times \left(1.35\times \frac{P_{dHyp}}{P_{aHyp}}-0.32\right)$	Hyperemic microcirculatory resistance corrected for collateral flow
Baseline resistance index (BRI)	$P_{dRest}\times {Tmn}_{Rest}$	Resting microcirculatory resistance
Resting flow velocity (RFV)	$\frac{1}{{Tmn}_{Rest}}$	Estimated resting flow velocity.
Hyperemic flow velocity (HFV)	$\frac{1}{{Tmn}_{Hyp}}$	Estimated hyperemic flow velocity
Coronary flow reserve (CFR)	$\frac{{Tmn}_{Rest}}{{Tmn}_{Hyp}}$	Hyperemic coronary flow velocity in relation to resting flow velocity
Microvascular resistance reserve (MRR)	$\frac{CFR}{FFR}\times \frac{P_{aRest}}{P_{aHyp}}$	Resting microcirculatory resistance in relation to hyperemic microcirculatory resistance, adjusted for the presence of epicardial lesions
Fractional flow reserve (FFR)	$\frac{P_{dHyp}}{P_{aHyp}}$	Distal coronary pressure in relation to aortic pressure during hyperemia

*P*_*aHyp*_ = aortic pressure during hyperemia; *P*_*aRest*_ = aortic pressure during rest; *P*_*dHyp*_ = distal coronary pressure during hyperemia; *P*_*dRest*_ = distal coronary pressure during rest; *Tmn*_*Hyp*_ = mean transit time of a saline bolus during hyperemia; *Tmn*_*Rest*_ = mean transit time of a saline bolus during rest.

IMR_c_ in the LAD artery was elevated at 41 U (normal ≤25 U), and MRR was 1.45 U(normal, >3.0 U), indicating increased coronary microvascular resistance equivalent to CMD.^3^^,^^5^ The coronary flow reserve was 1.0 U (normal, ≥3.0 U), indicating severely impaired coronary flow attributable to both the epicardial lesion and microvascular dysfunction (Figure 2A). The left circumflex artery showed atheromatosis with an FFR of 0.87, and the right coronary artery had generalized atheromatosis with a midsegment lesion (FFR, 0.80). Invasively measured LV end-diastolic pressure was normal (10 mm Hg).

## Management

The patient was removed from the interventional laboratory without intervention according to the COMTESS study protocol. A later multidisciplinary conference recommended percutaneous coronary intervention (PCI) for the LAD, to which the patient consented. She was prescribed aspirin and scheduled for LAD artery revascularization the following week.

### Days 5 to 29

The patient experienced recurrent rapid atrial fibrillation, effectively managed with beta-blocker therapy. Aspirin was temporarily discontinued because of worsening anemia requiring blood transfusions, leading to postponement of revascularization. Gastroscopy revealed multiple gastric ulcerations, treated with proton pump inhibitors. Fever and elevated infection markers prompted further investigation, and chest tomography revealed right-sided empyema with pleural effusion, which was drained. By day 29, her vital signs had normalized, and she was transferred to a cardiac ward in preparation for revascularization.

### Day 30

**Figure 3:**
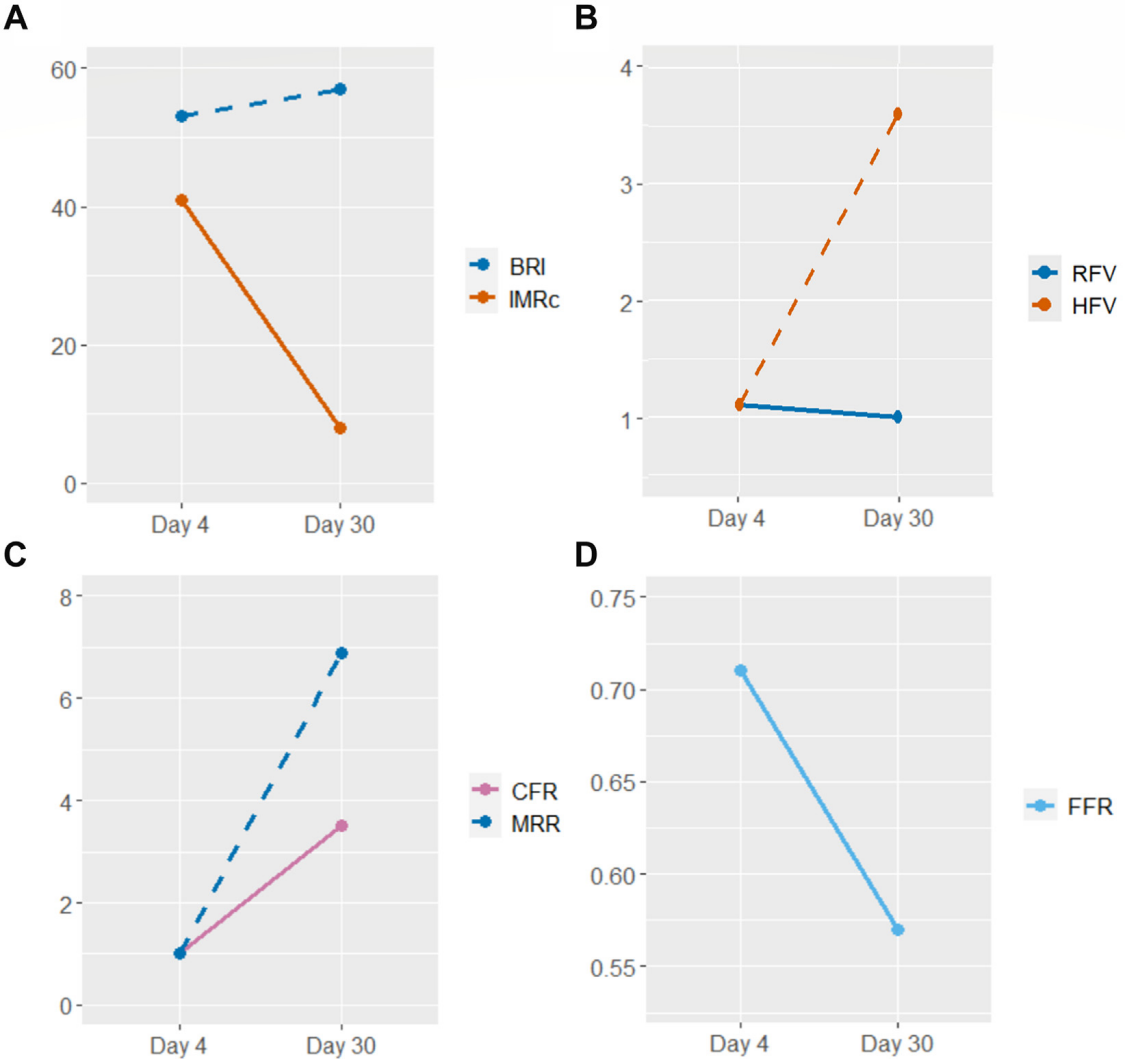
Changes in Coronary Velocity and Resistance Indices From Day 4 (Sepsis) to Day 30 (Recovery Phase, Pre-Percutaneous Coronary Intervention) (A) baseline resistance index (BRI), corrected index of microcirculatory resistance (IMR_c_), (B) resting flow velocity (RFV), hyperemic flow velocity (HFV), (C) coronary flow reserve (CFR), microvascular resistance reserve (MRR), and (D) fractional flow reserve (FFR).

**Figure 4:**
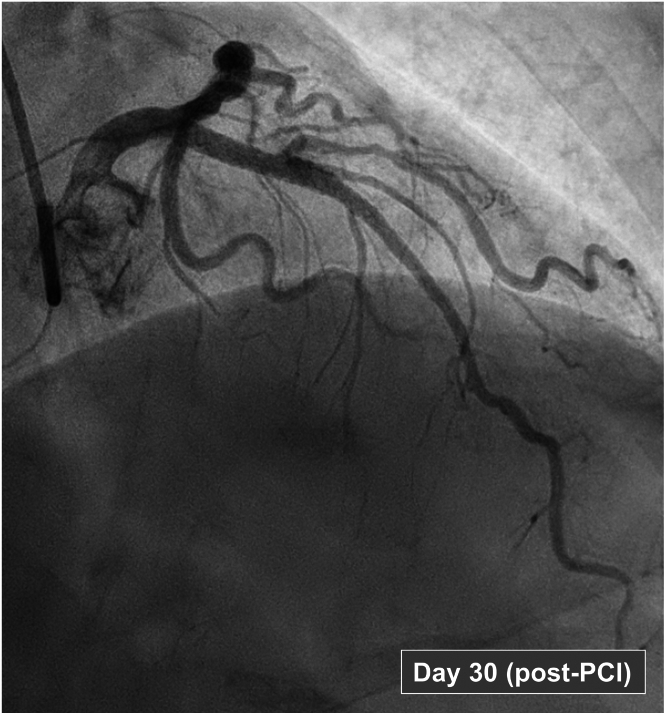
Coronary Angiography of the Left Anterior Descending Artery on Day 30 (Recovery Phase) After Scheduled PCI Initial dilation of the let anterior descending artery with a 3.0 balloon was followed by deployment of a 3.5 mm × 18 mm Xience ProS stent (Abbott) at 16 atm. The lesion was post-dilated with a 4.0 mm × 8 mm and a 3.5 mm × 12 mm noncompliant balloon for tapering. A distal stent edge dissection was noted necessitating the deployment of a 2.5 mm × 12 mm Xcience ProS stent at 18 atm. The overlap was postdilated with a 3.5 mm × 12 mm noncompliant balloon. A final optical coherence tomography run showed good stent apposition and expansion. PCI = percutaneous coronary intervention.

Microcirculatory measurements were performed before revascularization (Figure 2B). Resting flow remained essentially unchanged, whereas hyperemic flow, IMR_c_ and MRR showed significant improvement compared with the acute phase (Figures 2 and 3A to 3D). Although the proximal LAD artery lesion appeared angiographically unchanged from day 4, the FFR had decreased to 0.57, indicating previous underestimation of the epicardial obstruction secondary to CMD. The patient underwent PCI with 2 drug-eluting stents in the LAD artery following preloading with 600 mg of clopidogrel. The final angiogram is presented in Figure 4. Post-revascularization, the patient was prescribed long-term apixaban and atorvastatin, along with a 6-month course of clopidogrel.

## Outcome and Follow-Up

At the 1-month follow-up, the patient remained asymptomatic, with no reported cardiovascular events.

## Discussion

We report the case of a 67-year-old woman with pneumococcal pneumonia and septic shock, complicated by myocardial injury and dysfunction, as indicated by elevated hs-cTnT and NT-proBNP levels, new onset atrial fibrillation, a significant LAD artery lesion, and CMD. In sepsis, these findings are associated with worsened short- and long-term prognosis.^6^ Notably, sepsis-related CMD contributed to an underestimation of epicardial stenosis severity and was present alongside echocardiographic evidence of reduced systolic function assessed by global longitudinal strain, despite preserved LVEF.

IMR is used to diagnose CMD and is unaffected by epicardial artery status.^7^ Although data on serial IMR measurements during recovery from myocardial infarction are limited, an IMR_c_ of 41, as observed in this case, is markedly elevated, indicating significant microvascular injury.^8^ Typically, such high IMR levels in myocardial infarction correlate with 3-digit levels of hs-cTnT and prolonged resolution times.^8^ Sepsis-related hyperinflammatory coronary microvascular injury likely differs pathologically from the acute ischemic microvascular injury seen in myocardial infarction, and this difference highlights the need for further investigation into its implications for patient outcomes.

Studies of sublingual microcirculation in sepsis patients consistently demonstrate heterogeneous perfusion distribution within the same vascular region.^1^ This raises the hypothesis that sepsis-related CMD may induce microvascular injury with a nonuniform distribution, affecting only a proportion of vessels supplied by each epicardial artery. Echocardiography showed reduced systolic LV function and low LV stroke volume despite normal LVEF. Consequently, myocardial wall motion abnormalities may deviate from typical myocardial infarction patterns, thereby making these patterns subtler or more difficult to detect.

IMR uses adenosine to induce hyperemia, thus promoting endothelium-independent vasodilation.^7^ Although our results indicate CMD, this method cannot discern the specific contributions of endothelial damage, intravascular microthrombosis, or autoregulatory dysfunction. Studies have shown that microvascular inflammation can induce functional changes in endothelial and smooth muscle cells, leading to structural alterations, vascular remodeling, and coronary plaque destabilization.^9^ Furthermore, recent reports indicate that sepsis survivors face an increased risk of major cardiovascular events, so we cannot assume that transient CMD signifies complete recovery of the microvascular wall.^6^^,^^10^

We observed significant troponin fluctuations even in the presence of acute kidney failure. The patient’s initial dyspnea and chest pain were likely pneumonia related, although cognitive impairment during septic shock rendered anamnesis unreliable. Differentiating between type 1 and type 2 myocardial infarction is challenging, but high hyperemic resistance, modest hs-cTnT elevation, absence of regional wall motion disturbances on echocardiography, and rapid CMD recovery make type 1 myocardial infarction unlikely. The absence of apical ballooning and preserved or hyperkinetic basal segments argues against takotsubo syndrome, although low hs-cTnT and elevated NT-proBNP cannot exclude it earlier in the clinical course.

Multiple factors—including respiratory failure, underlying epicardial coronary artery disease, anemia, hypotension, shock, and CMD with high hyperemic coronary resistance—suggest an oxygen supply-demand mismatch, thus making type 2 myocardial infarction the most likely diagnosis.

## Conclusions

This case provides new insights into the involvement of CMD in sepsis, highlighting the need for further research to understand its implications for recovery and prognosis.

**Visual Summary:**
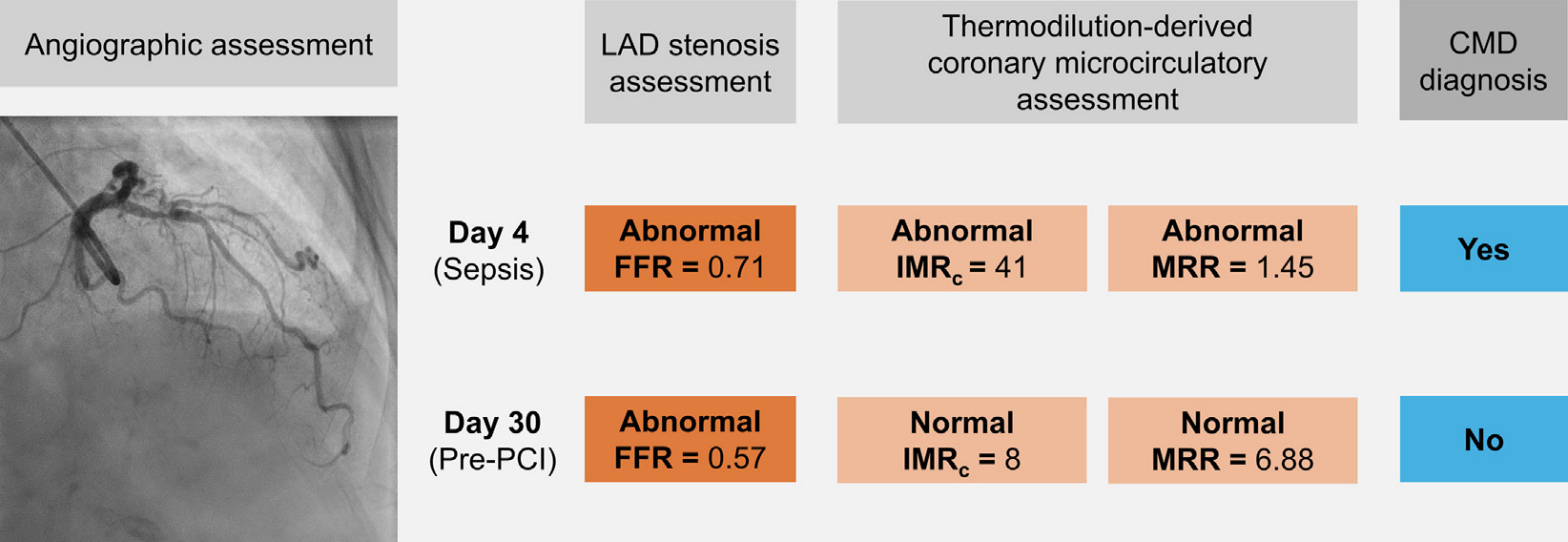
Resolution of Sepsis-Induced Coronary Microvascular Dysfunction

## Funding Support and Author Disclosures

The authors have reported that they have no relationships relevant to the contents of this paper to disclose.
